# Non-Invasive Electrotherapy in the Rehabilitation of Motor Sequelae and Spasticity Following Stroke: A Systematic Review

**DOI:** 10.3390/jcm15083085

**Published:** 2026-04-17

**Authors:** Mariola Lledò Amat, Marlene García-Quintana, Martin Vilchez-Barrera, Aníbal Báez-Suárez, Fabiola Molina-Cedrés, Rafael Arteaga-Ortiz, David Alamo-Arce, Raquel Medina-Ramirez

**Affiliations:** 1 Faculty of Health Sciences, University of Las Palmas de Gran Canaria, 35016 Las Palmas, Spain; mariola.lledo101@alu.ulpgc.es (M.L.A.); martin.vilchez@ulpgc.es (M.V.-B.); anibal.baez@ulpgc.es (A.B.-S.); fabiola.molina101@alu.ulpgc.es (F.M.-C.); rafael.arteaga@ulpgc.es (R.A.-O.); 2 Soc-Dig Research Group, University of Las Palmas de Gran Canaria, 35016 Las Palmas, Spain; danieldavid.alamo@ulpgc.es (D.A.-A.); raquel.medina@ulpgc.es (R.M.-R.)

**Keywords:** electric stimulation therapy, physical therapy, stroke, transcutaneous electric nerve

## Abstract

**Background/Objectives**: Stroke is a sudden neurological event caused by disrupted cerebral blood flow and represents a leading cause of acquired disability worldwide. Motor impairments and spasticity are among the most prevalent sequelae, significantly limiting functional independence and quality of life. Non-invasive electrotherapy has emerged as a complementary strategy in neurorehabilitation aimed at enhancing neuroplasticity and improving motor recovery. To systematically review current evidence regarding the effectiveness of non-invasive electrotherapy modalities in the rehabilitation of motor sequelae and spasticity following stroke, and to examine their theoretical and clinical rationale. **Methods**: A systematic literature review was conducted in accordance with PRISMA 2020 guidelines. The protocol was prospectively registered in the Open Science Framework (OSF). A comprehensive search was performed in Pubmed, Web of Science (WoS), and Scopus for studies published up to 14 November 2023, using the terms “Electric Stimulation Therapy” and “Stroke”. The methodological quality was assessed using the PEDro scale. The levels of evidence were classified according to the Oxford Centre for Evidence-Based Medicine criteria, and the risk of bias was evaluated using the Cochrane Risk of Bias tool (RoB 2). **Results**: Sixteen studies were included in the review. The analyzed interventions comprised neuromuscular electrical stimulation (NMES), transcutaneous electrical nerve stimulation (TENS), functional electrical stimulation (FES), neuromuscular electrical stimulation combined with transcranial magnetic stimulation (NMES + rTMS), transcranial direct current stimulation (tDCS), and afferent electrical stimulation (AES). Overall, the methodological quality of the included studies ranged from moderate to high, with PEDro scores between 6 and 9 out of 10. According to the Oxford Centre for Evidence-Based Medicine classification, most studies corresponded to level 1b evidence, while a smaller proportion were classified as level 2b. A risk of bias assessment using the Cochrane RoB 2 tool showed that the majority of the included studies presented a low risk of bias across most domains, although some concerns were identified in the domains of randomization and measurement in a limited number of trials. Across modalities, consistency within group improvement in motor function and spasticity was reported. However, between group comparisons with conventional rehabilitation were often inconsistent and did not consistently demonstrate superiority. The variability in stimulation parameters, intervention duration, and outcome measures further limited direct comparisons across studies. **Conclusions**: Non-invasive electrotherapy appears to be a safe and promising adjunct to conventional stroke rehabilitation. Nevertheless, further high-quality studies are required to clarify the underlying neurophysiological mechanisms and to establish standardized treatment protocols.

## 1. Introduction

The term ‘stroke’ refers to a sudden neurological deficit caused by an alteration in cerebral blood flow, whether ischemic or hemorrhagic in origin, leading to an interruption in the supply of oxygen and nutrients to brain tissue [1,2,3]. This condition is one of the leading causes of mortality worldwide and in Europe and is the leading cause of acquired neurological disability in adults, with a significant long-term impact due to the motor, sensory, cognitive and emotional sequelae experienced by survivors [2,3,4,5].

Among the most common sequelae after a stroke are motor deficits and spasticity, which considerably limit functionality, autonomy and quality of life. These alterations are associated with changes in cortical and spinal excitability, maladaptive reorganization of neural circuits and loss of descending inhibitory control, which compromises motor recovery and promotes the development of abnormal movement patterns [6,7].

Despite advances in conventional neurological rehabilitation, functional outcomes remain variable and, in many cases, insufficient to achieve optimal recovery. This situation has prompted the development of complementary therapeutic strategies aimed at enhancing neuroplasticity and facilitating the functional reorganization of the central nervous system after stroke [6,8,9,10,11]. Neuroplasticity after stroke is largely activity-dependent, requiring repeated and task-specific stimulation to promote adaptative cortical reorganization. Non-invasive neuromodulation techniques may enhance these mechanisms by modulating cortical excitability and facilitating synaptic plasticity, thereby potentially augmenting the effects of conventional rehabilitation.

In this context, non-invasive electrotherapy has emerged as a promising tool in the field of neurological rehabilitation. These techniques use electrical or electromagnetic stimuli to induce biological and physiological responses at the central and peripheral levels, modulating the neuronal excitability and promoting activity-dependent motor learning processes, thereby enhancing activity-dependent plasticity mechanisms involved in motor relearning [8,9,10,11,12].

Among the non-invasive electrotherapy modalities used in physiotherapy is neuromuscular electrical stimulation (NMES), which aims to induce muscle contractions to improve strength, prevent atrophy and facilitate motor activation, which is particularly relevant in patients with upper motor neuron involvement [9].

Functional electrical stimulation (FES) aims to restore voluntary movement by delivering electrical stimulation to peripheral nerves or muscles during functional tasks, thereby promoting task-specific motor relearning and improving motor performance.

On the other hand, transcutaneous electrical nerve stimulation (TENS), traditionally used for analgesic purposes, has been shown to have modulating effects on cortical and spinal excitability, which may contribute to reducing spasticity and improving motor performance in post-stroke patients [10]. Similarly, central neuromodulation techniques such as transcranial magnetic stimulation (TMS) act directly on the cerebral cortex, modulating neuronal excitability and promoting the cortical reorganization processes involved in functional recovery [6,8,9,10,11].

Transcranial direct current stimulation (tDCS) delivers low-intensity electrical currents to modulate cortical excitability, facilitating adaptative neuroplastic chances associated with motor recovery. In addition, transcutaneous spinal direct current stimulation (tsDCS) has been proposed as a method to influence spinal excitability and modulate descending motor pathways, potentially contributing to spasticity control [8,9,10,11].

Afferent electrical stimulation (AES), which targets sensory pathways, has also been investigated for its capacity to enhance sensorimotor integration and promote cortical reorganization in post-stroke rehabilitation [9,10,11].

Despite the growing body of literature supporting the use of non-invasive electrotherapy in stroke rehabilitation, there is no clear consensus regarding the comparative effectiveness of these modalities, the optimal stimulation parameters, or their specific impact on motor recovery and spasticity reduction. The substantial heterogeneity in study designs, intervention protocols, and outcome measures hampers direct comparison between techniques and limits evidence-based clinical decision-making. This lack of standardization underscores the need for a comprehensive rigorous synthesis of the current evidence [6,8,9,10,11].

Although several systematic reviews have evaluated the effects of specific non-invasive electrotherapy modalities such as NMES, FES, TENS, and tDCS, these studies have generally focused on individual techniques in isolation [7,10]. As a result, there is a lack of comprehensive syntheses that comparatively evaluate multiple electrotherapy modalities within a unified clinical framework. These techniques have been progressively introduced into stroke rehabilitation over recent decades, with growing interest in their potential to enhance neuroplasticity and functional recovery.

Furthermore, previous reviews have often addressed either motor recovery or spasticity separately, without integrating both outcomes into the context of neurorehabilitation. Therefore, the present review aims to provide a broader and more clinically relevant perspective by analyzing and comparing different non-invasive electrotherapy approaches and their effects on both motor function and spasticity in post-stroke patients.

Therefore, the primary aim of this systematic literature review was to evaluate the overall effectiveness of non-invasive electrotherapy modalities in improving motor function and reducing spasticity after stroke. A secondary aim was to provide a comparative overview of the different techniques used in clinical practice and to explore their potential role as adjunctive interventions in neurorehabilitation.

## 2. Materials and Methods

This systematic review was conducted in accordance with the Preferred Reporting Items for Systematic Reviews and Meta-Analyses guidelines [13]. Due to the high heterogeneity across studies in terms of interventions, outcome measures, and patient characteristics, a quantitative meta-analysis was not performed.

The completed PRISMA checklist is available in (Supplementary Materials PRISMA 2020 Checklist) and was prospectively registered in the Open Science Framework (OSF)—registration number: K3D42; DOI: 10.17605/OSF.IO/K3D42. This work is part of a doctoral thesis at the University of Las Palmas de Gran Canaria.

### 2.1. Search Criteria and Strategy

The review question was structured according to the PICO framework: Population (adult stroke patients), Intervention (non-invasive electrotherapy), Comparison (conventional rehabilitation or sham stimulation), and Outcomes (motor function and spasticity).

The search was performed using the following equation (“electric stimulation therapy”[MeSH Terms] AND “stroke”[MeSH Terms]) AND ((clinicaltrial[Filter] OR randomizedcontrolledtrial[Filter]) AND (2015/1/1:2023/11/14[pdat])).

### 2.2. Eligibility Criteria

We undertook a comprehensive review of the literature in the PubMed, Web of Science (WOS), and Scopus databases on the treatment of stroke sequelae, using the keywords ‘Electric Stimulation Therapy’ and ‘Stroke’, up to 14 November 2023.

The research question upon which we based this systematic review is ‘what non-invasive electrotherapy treatment techniques are currently used in patients with post-stroke motor sequelae and spasticity, and how effective are they, according to the existing scientific literature?’

### 2.3. Inclusion Criteria

Research articles on neurological pathology in humans involving people over the age of 18 affected by strokes were included. The studies had to evaluate post-stroke treatment using electrotherapy or compare it with other therapies, focusing on motor sequelae and spasticity. Articles dealing with different types of strokes and at different stages of their evolution were included.

The PEDro scale was used to assess the methodological quality of the included randomized controlled trials, as well as pilot studies following a controlled and randomized design. PEDro scores were considered during the interpretation of the findings, but studies were not excluded solely on the basis of these scores. Articles published between 2015 and 2023, available in English or Spanish, were included.

### 2.4. Exclusion Criteria

Studies addressing aphasia or other non-motor sequelae, studies conducted in pediatric populations, and studies conducted in healthy patients or patients with conditions other than stroke were excluded. Articles and studies based on invasive electrotherapy techniques were also excluded.

### 2.5. Study Selection and Data Extraction

Two independent reviewers screened all titles and abstracts for relevance and eligibility. Full-text articles meeting the inclusion criteria were subsequently assessed for final inclusion. Any discrepancies between reviewers were resolved through discussion and consensus. When necessary, a third reviewer was consulted to ensure methodological rigor.

Data extraction was performed independently by two reviewers, using a standardized data extraction form. The extracted information included the study design, sample size, participant characteristics (age, stroke type and chronicity), details of the electrotherapy intervention (modality and stimulation parameters such as frequency, intensity, pulse width, session duration, numbers of sessions, and electrode placement), comparison or control conditions, follow-up duration, outcome measures related to motor function and spasticity, and main findings.

### 2.6. Quality Assessment

The methodological quality of each study was assessed using the Physiotherapy Evidence Database (PEDro) scale, which evaluates internal validity and statistical reporting across ten items [14]. Each satisfied item (excluding item 1, which relates to external validity) received one point, with the total scores ranging from 0 to 10. Scores of 6 or higher were considered to be of a high quality.

The risk of bias was further assessed using the revised Cochrane Risk-of-Bias Tool for Randomized Trials (RoB 2) [15], which analyses five domains: the randomization process, deviations from intended interventions, missing outcome data, the measurement of outcomes, and the selection of reported results.

The overall strength of evidence was graded according to the Oxford Centre for Evidence-Based Medicine (CEBM) hierarchy [16], ranging from Level 1 (systematic reviews of randomized trials) to Level 5 (expert opinion). These three tools provided complementary perspectives on methodological rigor and evidential weight.

## 3. Results

### 3.1. Study Selection

The search strategy used yielded 496 articles, and four articles were found from other sources (OSF registration). After analyzing the search results, three articles were excluded because they were duplicates. After analyzing the titles, an initial screening was carried out and 149 articles were eliminated. The abstracts of 347 publications were then read, from which 67 documents were selected.

**Figure 1:**
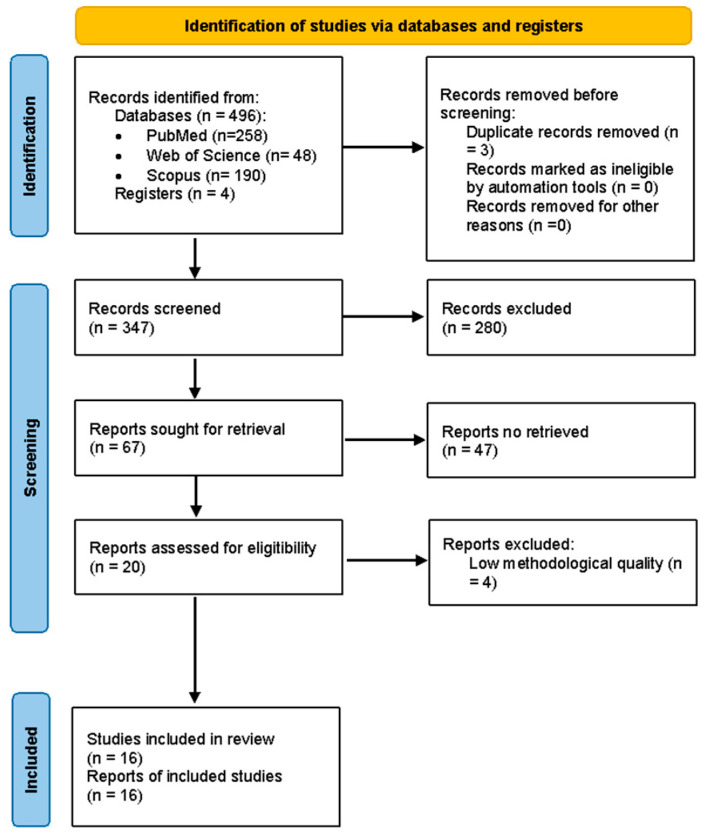
PRISMA 2020 flow diagram of study selection. PRISMA 2020 flow diagram detailing the study selection process. This diagram summarizes the identification, screening, eligibility, and inclusion phases of the systematic review. It was adapted from the PRISMA 2020 statement (Page et al., 2021 [13]) and constructed using the official template.

After applying the inclusion criteria, we obtained 20 studies for full reading and, after applying the Oxford Centre for Evidence-Based Medicine (OCEBM) classification, the PEDro scale and the Rob II scale, we chose the 16 that are included and analyzed in this review (Figure 1).

### 3.2. Methodological Quality Assessment

The methodological quality of the included studies was evaluated using the PEDro scale (Table 1). This scale assesses the internal validity and statistical reporting across ten items. Each criterion was met, except for Item 1, which refers to external validity and scored one point, yielding a total score between 0 and 10. Studies with scores ≥ 6 were classified as being of a high methodological quality.

The overall strength of the evidence was graded according to the Oxford Centre for Evidence-Based Medicine hierarchy (Table 1), in which Level 1 represents systematic reviews of randomized trials and Level 5 represents an expert opinion.

To complement this quantitative evaluation, the risk of bias was assessed using the revised Cochrane Risk of Bias tool (RoB 2), which covers five domains: randomization, deviations from intended interventions, missing outcome data, measurement of outcomes, and selection of reported results. Each domain was judged as having a low risk, some concerns, or a high risk of bias (Table 2).

### 3.3. Summary of Results

Sixteen articles were analyzed, including eleven randomized controlled trials and five pilot studies with a controlled and randomized design, which investigated the treatment of 474 adult patients. Of these patients, 279 were men and 195 were women. Table 3 shows the procedures, and Table 4 shows the parameters used in electrotherapy techniques. The time since stroke and baseline motor impairment varied considerably across studies and were not consistently reported, with some studies including chronic patients (>6 months), while others included acute or subacute populations. The baseline motor deficits were assessed using different scales (e.g., FMA, MAS), limiting the direct comparability. Based on the techniques analyzed in each article, the results obtained present the following information.

#### 3.3.1. NMES

The pilot study by Baricich et al. (2019) investigated the effect of neuromuscular electrical stimulation (NMES) in hemiplegic patients with clubfoot [17]. Thirty patients were divided into two groups: one received NMES on the muscles injected with botulinum toxin and on the antagonist muscles, while the other group only received NMES on the injected muscles. Both groups showed significant improvements in muscle tone and gait analysis, but there were no significant differences between groups.

#### 3.3.2. NMES vs. TENS

A randomized pilot study conducted by Yen et al. (2019) in 42 adult patients investigated the differences between two interventions with TENS and NMES alongside standard early rehabilitation, or standard early rehabilitation alone [26]. The results only showed a significant improvement in the TENS group compared to the early rehabilitation alone group.

#### 3.3.3. NMES + rTMS

Etoh et al. (2019) conducted a cross-over RCT with 20 stroke patients to investigate whether NMES can enhance the effects of rTMS and repetitive facilitation exercises (RFE) on hemiparetic hand function [21]. All participants received 1 Hz rTMS to the unaffected motor cortex (10 min) and RFE (60 min); the experimental group received NMES and the control group received sham NMES. The results suggest that the combination of NMES and rTMS could enhance the positive effects of RFE on the functionality and spasticity of the affected upper limb.

#### 3.3.4. tDCS

The pilot study by Cunningham et al. (2015) with 12 patients divided into two groups proposed to investigate the effectiveness of ipsilesional anodal tDCS in combination with movement restriction therapy [30]. The results show significant improvements in function and dexterity in patients who received tDCS. An increase in excitability was also observed in the contralesional hemisphere, rather than the ipsilesional hemisphere.

Allman et al. (2016) conducted an RCT with 24 stroke patients without primary motor cortex involvement, divided into two groups (real and sham stimulation), to evaluate the effect of ipsilesional anodal tDCS [29]. The results showed persistent improvements at 3 months in the real tDCS group, demonstrating greater activity in the ipsilesional motor and premotor cortex during movement of the affected hand. In addition, structural magnetic resonance imaging revealed an increase in gray matter volume in these cortical areas after anodal tDCS.

The pilot study by Straudi et al. (2016) evaluates the effects of bilateral tDCS combined with robot-assisted therapy on the upper limbs in 23 stroke patients [27]. Both groups (real and sham stimulation) showed significant improvements, but no significant differences between them. However, patients with chronic and subcortical stroke benefited more than those with acute and cortical stroke.

Hamoudi et al. (2018) conducted an RCT with 56 chronic post-stroke patients with mild impairment to evaluate the effectiveness of tDCS combined with learning a new motor skill [28]. The patients were divided into two groups (real and sham tDCS). Both groups improved in the new task, but the real tDCS group showed a significantly greater increase in motor skill. No significant differences were found between groups in the long-term retention of the new skill.

Beaulieu et al. (2019) conducted a parallel two-arm pilot trial with 14 patients, in which upper limb-focused resistance training was combined with real and sham tDCS [19]. The results indicate that both groups improved, but with no significant changes between groups.

The study by Koganemaru et al. (2019) included 11 patients in the chronic phase of stroke in a single-blind crossover trial, comparing the application of real and simulated tDCS, associated with NMES, during treadmill walking [22]. The results showed that walking speed increased significantly after a single session of real tDCS, but not with simulated stimulation. With repeated interventions, significant improvements were observed in speed, timed test performance, balance, and greater joint flexion of the paretic limbs during walking.

Mazzoleni et al. (2019) conducted an RCT with 40 patients in the subacute post-stroke phase to evaluate the effectiveness of combining tDCS and robot-assisted wrist rehabilitation, comparing it to robotic training alone [23]. It was observed that all clinical outcome measures, except for the modified Ashworth Scale for spasticity, showed significant improvements after treatment in both groups, but with no significant differences between them.

The publication by Doost et al. (2019) presents a crossover RCT; it includes 21 chronic patients with hemiparesis who undergo sessions with real or simulated bihemispheric tDCS alongside training in a cooperative bimanual task (CIRCUIT) [20]. The results show that learning bimanual motor skills did not improve with the application of tDCS.

The publication by Picelli et al. (2019) follows an RCT to compare the effectiveness of two cerebellar tDCS protocols together with transcutaneous spinal stimulation in robotic gait training in patients with chronic supratentorial stroke [24]. It included 40 patients, and the results show that both interventions led to improvements in the 6 min walk test, but there were no significant improvements between groups.

The RCT by Alisar et al. (2020) included 32 patients divided into two groups [18]. All received physiotherapy and occupational therapy for three weeks; in addition, group 1 received bihemispheric tDCS and group 2 received sham tDCS. The results showed a significant improvement in motor function in the group with real tDCS compared to the other group, with better results observed in chronic patients.

#### 3.3.5. AES

Another study included in this review is an RCT conducted by Lee and Lee (2019), which grouped 30 patients to study the effect of AES combined with mirror therapy on motor function, balance, and gait in chronic stroke [25]. The results show that in the real intervention group, there were significant improvements in the elements analyzed.

#### 3.3.6. FES

The RCT by Kim and Jang (2021) studied the effect of mirror therapy combined with electromyography-activated FES in 60 patients with chronic stroke [31]. They were divided into three groups, and the results did show significant improvements in balance and gait in the intervention group that combined FES with mirror therapy. 

Finally, the study by Dantas et al. (2023) compared the effects of treadmill training with functional electrical stimulation (TT-FES) versus treadmill training (TT) alone in 28 stroke patients [32]. The results showed that the group that started with TT-FES had improvements in their mobility, balance, coordination of non-paretic limbs, and endurance. Sensory-motor function improved in all patients, regardless of the order of training. Coordination of paretic limbs improved only in the group that received TT-FES after TT.

## 4. Discussion

The present systematic review aimed to synthesize the current evidence regarding the effectiveness of non-invasive electrotherapy modalities in the rehabilitation of motor sequelae and spasticity following stroke. Overall, the findings suggest that these techniques may provide beneficial effects on motor recovery and spasticity management, particularly when applied as complementary interventions alongside conventional rehabilitation. However, their effectiveness appears to depend on multiple factors, including stimulation parameters, stroke chronicity, lesion location, and the integration of electrotherapy with active motor training.

These findings should be interpreted with caution, as demographic factors such as sex, age, and population characteristics were not consistently reported across studies (see strengths and limitations section). The substantial heterogeneity observed across studies in terms of intervention protocols, patient characteristics, and outcome measures limited the feasibility of conducting a meaningful meta-analysis.

The findings of this review should be interpreted primarily in terms of overall effectiveness, rather than direct superiority between modalities, given the heterogeneity of the included studies.

### 4.1. Peripheral Electrotherapy Modalities

Peripheral stimulation techniques, including neuromuscular electrical stimulation (NMES), functional electrical stimulation (FES), transcutaneous electrical nerve (TENS), and afferent electrical stimulation (AES), demonstrated potential benefits in improving motor function and reducing spasticity when incorporated into rehabilitation programs [25,31,32]. NMES has been proposed to facilitate motor unit recruitment and enhance reciprocal inhibition mechanisms, which may contribute to improved neuromuscular control [17]. Nevertheless, several studies reported inconsistent outcomes, particularly when NMES was applied as a stand-alone intervention, suggesting that its effectiveness may be enhanced when combined with task-oriented rehabilitation strategies [17,21,26].

Similarly, FES has shown promising results in improving gait, balance, and functional mobility, especially when synchronized with voluntary movement or functional tasks [32,33]. These findings support the concept of activity-dependent plasticity, in which repeated motor practice combined with peripheral stimulation may strengthen neural pathways involved in motor control [31,32].

TENS and AES appear to influence sensory afferent pathways and may modulate cortical excitability through enhanced sensorimotor integration [18,19,20,21,22,23,24,25,26,27,28,29,30]. These mechanisms may contribute to improvement in motor performance and spasticity control. However, considerable variability in the stimulation parameters, treatment duration, and outcome measures across studies limits the comparability of the findings and highlights the need for more standardized protocols [18,19,20,21,22,23,24,25,26,27,28,29,30,31,32].

### 4.2. Central Neuromodulation and Interhemispheric Balance

Central Neuromodulation techniques such as transcranial direct current stimulation (tDCS), neuromuscular electrical stimulation combined with transcranial magnetic stimulation (NMES + rTMS), and transcutaneous spinal direct current stimulation (tsDCS) aim to modulate cortical and spinal excitability to facilitate motor recovery stroke [18,19,20,22,23,24,27,28,29,30]. Although peripheral and central electrotherapy modalities are presented separately for conceptual clarity, it should be noted that their mechanisms are not independent, as both interact through bidirectional pathways involving cortical and spinal networks. These approaches are frequently based on the interhemispheric imbalance model, which proposes that decreased excitability of the ipsilesional hemisphere and increased inhibitory activity from the contralesional hemisphere contributed to persistent motor deficits following stroke [17,21,22,24,26]. It should be noted that the interhemispheric imbalance model may not apply uniformly to all post-stroke patients, as individuals with similar clinical presentations can exhibit heterogeneous neurophysiological patterns. This variability may contribute to the inconsistent results observed across studies. However, in clinical practice, non-invasive electrotherapy is primarily guided by functional and symptomatic outcomes rather than individualized neurobiological profiles. It should be noted that polarity-dependent effects (e.g., anodal vs. cathodal stimulation) are primarily described in transcranial direct current stimulation and should not be generalized across all electrotherapy modalities, as different techniques rely on distinct neurophysiological mechanisms [28,30].

Interventions targeting this imbalance either attempt to enhance excitability in the affected hemisphere or to inhibit activity in the contralesional hemisphere. Several studies included in this review reported improvements in motor performance, gait, and functional outcomes following the application of tDCS or TMS, particularly when these techniques were combined with task-specific motor training, robotic-assisted therapy, or constraint-induced movement therapy [19,20,22,23,24,25,28].

Nevertheless, some trials reported similar improvements in both the real and sham stimulation groups, suggesting that the additive effect of neurodomulation may depend on patient-specific factors such as stroke chronicity, lesion location, and baseline functional status [19,20,24]. Evidence also indicates that individuals with chronic stroke may respond differently to neuromodulation compared to those in subacute stages, emphasizing the importance of individualized rehabilitation approaches [25,28]. Interindividual variability in responsiveness to neuromodulation may also contribute to the lack of significant between-group differences observed in several trials, as group-level analyses may mask differential individual effects.

### 4.3. Clinical Implications

From a clinical perspective, the available evidence suggests that non-invasive electrotherapy should be considered an adjunctive strategy, rather than a replacement for conventional rehabilitation [18,19,20,21,22,23,24,25,26,27,28,29,30,31,32]. In this context, the combination of central and peripheral electrotherapy modalities may offer potential synergistic effects by targeting different levels of the neuromotor system, including cortical excitability and peripheral motor activation. However, the underlying mechanisms of these combined approaches remain incompletely understood, and their effectiveness may depend on complex interactions between stimulation modalities and patient-specific factors. The most consistent improvements were observed when electrotherapy modalities were combined with active, task-oriented interventions that promoted motor learning and neuroplastic adaptation [19,20,31].

Furthermore, patient selection appears to play a critical role in determining treatment effectiveness. Factors such as time since stroke onset, lesion characteristics, and baseline motor function may influence responsiveness to neuromodulation and peripheral stimulation techniques [25,28]. Consequently, individualized treatment protocols that integrate clinical assessment with neurophysiological considerations may represent a promising approach for optimizing rehabilitation outcomes.

Although consistent improvements in motor function and spasticity were observed across several non-invasive electrotherapy modalities, these effects were predominantly reported as within-group changes. In contrast, group comparisons with conventional rehabilitation were often inconsistent or failed to demonstrate clear superiority.

This suggests that non-invasive electrotherapy should be primarily considered as an adjunctive intervention, rather than a standalone or superior therapeutic approach. From a clinical perspective, this distinction is important, as it supports the integration of electrotherapy into multimodal rehabilitation programs, rather than its use as a replacement for conventional therapies. In addition, the variability in stimulation parameters, intervention duration, and outcome measures across studies limits direct comparability and further complicates the interpretation of treatment efficacy.

Additionally, factors such as post stroke fatigue may influence patient engagement and response to rehabilitation interventions. Recent evidence suggests that different fatigue subtypes may differentially impact rehabilitation outcomes, highlighting the need for individualized therapeutic approaches [33].

These findings are consistent with recent consensus-based neurorehabilitation frameworks, which conceptualize post-stroke rehabilitation as a multidimensional process targeting not only neurophysiological mechanisms, but also functional performance, activity, and participation, in line with the International Classification of Functioning, Disability and Health (ICF) model [34].

### 4.4. Strengths and Limitations

This review has several limitations that should be acknowledged. First, the heterogeneity in study designs, stimulation protocols, outcome measures, and patient characteristics precluded quantitative meta-analysis and limited direct comparison between electrotherapy modalities. Variability in stimulation parameters, including frequency, intensity, electrode placement, and session duration, further complicates the identifications of optimal treatment protocols.

One limitation of this review is that the literature search was conducted up to November 2023. Given the rapid development of non-invasive electrotherapy research, more recent studies may not have been included. Therefore, the findings should be interpreted within the context of the available evidence at the time of the search.

An important limitation of the present review was related to demographic characteristics of the included populations. Across the analyzed studies, a predominance of male participants was observed; however, sex was not consistently examined as a moderating factor. This is particularly relevant, given that sex-related differences in cortical excitability and neuroplasticity may influence the response to non-invasive electrotherapy interventions. Another limitation of the current evidence is the scarce use of neuroimaging or neurophysiological measures, with most studies relying on clinical and functional outcomes. While these measures are essential for assessing rehabilitation effectiveness, the integration of neuroimaging techniques may provide additional insight into the mechanisms underlying treatment response and interindividual variability.

The included studies were conducted across different geographic regions, including Europe, East Asia, and North America, yet the reporting of ethnicity and other population-specific characteristics was limited. Age distribution was also not systematically analyzed, despite its well established influence on neuroplasticity and functional recovery following stroke. These factors may limit the generalizability of the findings and suggest that future research should incorporate more detail reporting and analysis of demographic variables to better understand population-specific responses to electrotherapy interventions.

Additionally, several of the included studies presented relatively small sample sizes and moderate methodological quality [19,20,22,23,24,28], which may reduce the robustness of the reported findings. Differences in the stroke chronicity and lesion location also contribute to variability in treatment responsiveness [18,19,20,22,23,24,25,28]. Moreover, long-term follow-up data were limited in many studies, preventing definitive conclusions regarding the sustainability of treatment effects. Finally, publication bias cannot be completely excluded, as studies reporting positive outcomes may be more likely to be published.

Despite these limitations, this review also presents important strengths. The protocol was prospectively registered in the Open Science Framework (OSF), enhancing transparency and methodological rigor. The review was conducted according to the PRISMA 2020 guidelines [13] and included a comprehensive search strategy across multiple databases. Furthermore, methodological quality, level of evidence, and risk of bias were systematically assessed using validated tools, including the PEDro scale [14], Oxford Centre for Evidence Based Medicine criteria [16], and the RoB 2 tool [15]. Unlike previous reviews focusing on a single modality, this study provides a comparative synthesis of multiple non-invasive electrotherapy techniques, integrating clinical outcomes with neurophysiological mechanisms to offer a broader and clinically relevant perspective.

The interpretation of the findings should also consider the methodological quality of the included studies. Although the majority of studies were assessed as having a low risk of bias across most domains, several studies presented some concerns, particularly in relation to the randomization process and outcome measurement. These methodological limitations may influence the reliability of the reported effects and should be taken into account when interpreting the results.

Therefore, while the overall findings suggest beneficial effects of non-invasive electrotherapy, the strength of the evidence may vary depending on the methodological rigor of the individual studies.

### 4.5. Future Research Directions

Future research should aim to address the methodological heterogeneity observed across studies by developing standardized stimulation protocols and clearly defined outcome measures. Large-scale randomized controlled trials with adequate sample sizes and long-term follow-up are needed to clarify the durability of treatment effects [17,18,19,20,21,22,23,24,25,26,27,28,29,30,31,32].

In addition, further research should explore the neurophysiological mechanisms underlying electrotherapy-induced recovery using neuroimaging and electrophysiological techniques. The identification of biomarkers associated with treatment responsiveness may help to personalize rehabilitation strategies and optimize patient selection.

Comparative studies evaluating different electrotherapy modalities and their combination with task-specific training or technological interventions, such as robotic-assisted rehabilitation, may also provide valuable insight into the most effective therapeutic approaches.

## 5. Conclusions

The available evidence suggests that non-invasive electrotherapy modalities including NMES, TENS, NMES + rTMS, tDCS, tsDCS, AES, and FES may be associated with improvements in motor function and spasticity in patients following stroke, particularly when applied as adjuncts to conventional rehabilitation. However, the results remain variable, and consistent superiority over conventional approaches has not been clearly demonstrated. However, the effectiveness of these interventions remains influenced by several factors, including the stimulation parameters, stroke chronicity, lesion characteristics, and patient-specific neurophysiological conditions. The heterogeneity observed across studies highlights the need for greater standardization of stimulation protocols and treatment parameters.

Future research should prioritize large-scale randomized controlled trials with standardized stimulation protocols and long-term follow-up in order to better understand the neurophysiological mechanism underlying electrotherapy-induced recovery and to support the development of evidence-based clinical guidelines.

## Figures and Tables

**Table 1:** Quality assessment of the included studies, using the PEDro and Oxford scales.

Article	PEDro Scale											Total	Oxford Scale
	I.1	I.2	I.3	I.4	I.5	I.6	I.7	I.8	I.9	I.10	I.11		
Baricich et al. (2019) [17]	YES	1	1	1	1	0	0	1	1	1	0	7/10	2b
Alisar et al. (2020) [18]	YES	1	1	1	1	0	1	0	1	1	1	8/10	1b
Beaulieu et al. (2019) [19]	YES	1	1	1	1	0	1	1	1	1	1	9/10	1b
Doost et al. (2019) [20]	YES	1	1	0	1	0	1	1	1	1	1	8/10	1b
Etoh et al. (2019) [21]	YES	1	1	1	1	0	1	1	1	1	1	9/10	1b
Koganemaru et al. (2019) [22]	YES	1	1	1	1	0	0	1	1	1	1	8/10	2b
Mazzoleni et al. (2019) [23]	YES	1	1	1	1	0	0	1	0	1	1	7/10	1b
Picelli et al. (2019) [24]	YES	1	1	1	0	0	1	1	1	1	1	8/10	2b
Lee et al. (2019) [25]	YES	1	1	1	1	0	1	1	1	1	1	9/10	1b
Yen et al. (2019) [26]	YES	1	1	1	0	0	1	1	0	1	1	7/10	2b
Straudi et al. (2016) [27]	YES	1	1	1	1	0	1	1	1	1	1	9/10	1b
Hamoudi et al. (2018) [28]	YES	1	1	1	0	0	1	1	0	1	1	7/10	1b
Allman et al. (2016) [29]	YES	1	1	1	1	0	1	1	0	1	1	8/10	1b
Cunningham et al. (2015) [30]	YES	1	1	0	1	0	1	1	1	1	1	8/10	1b
Kim and Jang (2021) [31]	YES	1	1	1	0	0	0	0	1	1	1	6/10	1b
Dantas et al. (2023) [32]	YES	1	0	1	0	0	1	1	0	1	1	6/10	1b

Table created by authors. Columns I.1–I.11 correspond to the individual items of the PEDro scale. Column I.1 is scored as 0 = no and 1 = yes.

**Table 2:** Risk of bias analysis. RoB 2.

Author/Year	D1 Randomization	D2 Deviations	D3 Missing Data	D4 Measurement	D5 Selection	Overall Risk
Alisar et al. (2020) [18]	**+**	**+**	**−**	**+**	**+**	**−**
Baricich et al. (2019) [17]	**−**	**−**	**+**	**+**	**+**	**−**
Beaulieu et al. (2019) [19]	**+**	**+**	**+**	**+**	**+**	**+**
Straudi et al. (2016) [27]	**−**	**+**	**+**	**+**	**+**	**−**
Cunningham et al. (2015) [30]	**+**	**+**	**+**	**+**	**+**	**+**
Doost et al. (2019) [20]	**+**	**+**	**+**	**+**	**+**	**+**
Etoh et al. (2019) [21]	**−**	**+**	**+**	**+**	**+**	**−**
Kim & Jang (2021) [31]	**−**	**×**	**+**	**−**	**+**	**×**
Allman et al. (2016) [29]	**+**	**+**	**+**	**+**	**+**	**+**
Koganemaru et al. (2019) [22]	**−**	**−**	**+**	**−**	**+**	**−**
Mazzoleni et al. (2019) [23]	**−**	**−**	**+**	**−**	**+**	**−**
Cunningham et al. (2015) [30]	**+**	**+**	**+**	**+**	**+**	**+**
Lee et al. (2019) [25]	**+**	**+**	**+**	**+**	**+**	**+**
Picelli et al. (2019) [24]	**+**	**+**	**+**	**+**	**+**	**+**
Hamoudi et al. (2018) [28]	**+**	**+**	**+**	**+**	**+**	**+**
Yen et al. (2019) [26]	**+**	**−**	**+**	**+**	**+**	**+**

Table created by authors. Risk ratings: low (+), some concerns (−) and high (×). A low rating denotes minimal risk of bias; ‘some concerns’ indicates a moderate risk that could affect result validity; and a high rating indicates substantial risk that may compromise reliability. The colors used in the table are green (low risk), yellow for some concerns, and red for high risk.

**Table 3:** Summary of intervention procedures, study design, and outcomes of included studies.

	Sample (n)	Methodology and Intervention	Assessment (Variables/Scales)	Results
**NMES**				
**Baricich** **2019** [17]	30 hemiplegic patients with spastic foot drop	Single-blind RCT. **G1**: (n = 15). 1 NMES session on injected agonist muscles. 5 NMES session on antagonist muscles. **G2:** (n = 15). 1 NMES session on injected agonist muscles. **Both groups:** 10 daily 60 min physiotherapy sessions. 2 weeks (5 days/week).	10 MWT. MAS. pROM. Antero tibial FM. 2 MWT. V_0_: Before treatment. V_1_: 10 days. V_2_: 20 days. V_3_: 90 days.	G1-G2: V_1_: Significant reduction in muscle tone and an increase in ankle ROM. V_2_ and V_3_: Significant increase in both groups in 10 MWT and 2 MWT. G1-G2: No significant differences.
**NMES vs. TENS**				
**Yen** **2019** [26]	42 patients with first stroke between the ages of 20 and 80 were admitted to the stroke center within 24 h of stroke onset.	Randomized controlled pilot study blinded by the evaluator. **G1:** (n = 13). Transcutaneous nerve stimulation and standard early rehabilitation. **G2:** (n = 13). Neuromuscular electrical stimulation and standard early rehabilitation. **G3**: (n = 14). Standard early rehabilitation. **Three groups:** 10 sessions (30 min per day, 5 days/week, for two weeks).	Postural assessment scale for stroke patients. Measure of functional independence. Three mobility milestones: sitting for 5 min, standing for 1 min, and walking ≥ 50 meters. V_0_: Before treatment. V_1_: 2 weeks. V_2_: 4 weeks.	G1-G2-G3: Significant improvements in all items within each group. G1-G2-G3: Significant differences in the Postural Assessment Scale for stroke patients and in functional independence in G1 compared to G3. Significant improvement in walking > 50 m independently in G1 and G2 compared to G3.
**NMES + rTMS**				
**Etoh** **2019** [21]	20 patients with hemiparesis.	Double-blind, crossover RCT. **G1:** (n = 10). Real NMES and simulated NMES. **G2**: (n = 10). Simulated NMES + NMES real. **Both groups**: 20 sessions over 4 weeks (5 days/week) of NMES (real or sham) for 10 min, simultaneously with rTMS in the unaffected motor cortex and RFE for 60 min.	FMA. ARAT. BBT. MAS. V_0_: Before treatment. V_1_: 2 weeks.	G1-G2: FMA and ARAT improved significantly. G1-G2: Significant improvements in BBT and EAM for wrist and finger only in real NMES.
**tDCS**				
**Cunningham** **2015** [30]	Patients with chronic stroke.	Randomized, double-blind pilot clinical study. **G1:** (n = 6). Anodal tDCS in ipsilesional upper motor areas. **G2:** (n = 6). Sham tDCS. **Both groups**: tDCS (real or sham) and movement restriction therapy for 30 min, twice a day, 3 days/week for 5 weeks (30 sessions).	Functional assessment. Neurophysiological assessment. V_0_: Before treatment. V_1_: 5 weeks.	G1: Only significant improvements in V_1_ in G1. G1-G2: In G1, significant improvements in function and dexterity compared to G2, and an increase in excitability of the contralesional hemisphere, rather than the ipsilesional hemisphere.
**Allman** **2016** [29]	24 patients (>6 months since first unilateral stroke without direct involvement of the primary motor cortex).	RCT. **G1**: (n = 11). Real anodal tDCS. **G2:** (n = 13). Simulated anodal tDCS. **Both groups**: 9 sessions of 1 h of tDCS (real or simulated) and motor training on consecutive days.	ARAT. WMF. FME-UE. Functional magnetic resonance imaging. Structural magnetic resonance imaging. V_0_: Before treatment. V_1_: 1 week. V_2_: 1 month. V_3_: 3 months.	G1-G2: In G1, significant improvements in V_3_ in all variables studied. In G2, no significant improvements were obtained in ARAT, WMF, or FME-UE. G1-G2: Functional MRI showed greater activity during movement of the affected hand in the ipsilesional motor and premotor cortex in G1 compared to G2. Structural MRI revealed intervention-related increases in gray matter volume in cortical areas, including the ipsilesional motor and premotor cortex in G1, but not in G2.
**Straudi** **2016** [27]	23 stroke patients.	Pilot study. **G1**: (n = 12). Robot-assisted therapy (RAT) + real tDCS. **G2**: (n = 11). RAT + sham-tDCS. **Both groups**: 10 sessions (5 sessions/week), two weeks.	FME: Upper extremity (UE). BBT. MAL. V_0_: Before treatment. V_1_: 3 weeks.	G1-G2: significant improvement in FMA-UE. BBT and FMA only showed significant improvements in G1. G1-G2. No significant differences in FMA. Significant improvements according to stroke type and duration, showing that stroke duration (acute versus chronic) and type (cortical versus subcortical) modify the effect of tDCS and RAT on motor function a significant improvement FMA-UE.
**Hamoudi** **2018** [28]	50 patients with chronic stroke with mild impairment.	RCT. **G1**: (n = 18). tDCS (anode over ipsilesional M1 and cathode on contralesional forehead). **G2**: (n = 18). Simulated tDCS. **G3**: (n = 14). No treatment. **G1 and G2**: tDCS (real or simulated) and sequential isometric pinch strength visual task for 1 h, 5 days a week, 1 week.	Learning (online and offline during the training period) and retention of a new motor skill. Generalization to untrained tasks. V_0_: Before treatment. V_1_: 8 days. V_2_: 29 days. V_3_: 57 days. V_4_: 85 days. V_5_: 113 days.	G1-G2-G3: All groups showed significant improvements in learning new motor skills. All groups applied the new motor skills to other tasks. G1-G2-G3: G1 showed significant improvements in motor skill learning (mainly in the online stage) compared to G2 and G3. There were no significant changes between groups in terms of long-term retention or generalization.
**Beaulieu** **2019** [19]	14 adults with supratentorial stroke.	Randomized controlled pilot trial, assessor-blinded. **G1**: (n = 7). Real tDCS. **G2**: (n = 7). Simulated tDCS. **Both groups**: 12 sessions of 60 min upper limb resistance training over 4 weeks (3 days/week).	FME. Box and block test. Wolf motor function test. Grip strength. MAS. Motor activity recording. V_0_: Before treatment. V_1_: 4 weeks.	G1-G2: Both groups improved clinical outcome measures. G1-G2: No significant differences.
**Koganemaru** **2019** [22]	11 patients with chronic stroke.	Single-blind, cross-over RCT. **G1**: 2 sessions (1 week between sessions). 1 session of oscillatory transcranial direct current stimulation over the affected area of the foot M1 and 1 session of sham stimulation during treadmill walking. Ankle dorsiflexion was assisted by electrical neuromuscular stimulation in real and simulated conditions.	10MWT. TUG. 6MWT. MAS. Mini-Balance. EVA. V_0_: Before stimulation. V_1_: After stimulation.	G1-G2: Significant improvements in 10MWT, TUG, 6MWT, Mini-balance and MAS if the intervention is applied repeatedly. G1-G2: Significant improvements in TUG are obtained with a single intervention.
**Mazzoleni** **2019** [23]	40 patients with subacute stroke.	Single-blind RCT. **G1:** (n = 20). Real tDCS. **G2:** (n = 20). Simulated tDCS. **Both groups**: 30 sessions of stimulation (real or simulated) and robotic wrist rehabilitation over 6 weeks (5 sessions/week).	Upper limb FME. MAS. Motor skills index. BBT. Wrist kinematic parameters measured by the robotic system (FE, PS, AA). V_0_: Before treatment. V_1_: 6 weeks.	G1-G2: Significant improvement in all clinical outcome measures except MAS. G1-G2: No significant differences between groups.
**Doost** **2019** [20]	21 patients with chronic stroke.	Double-blind, cross-over RCT. **G1:** (n = 10). tDCS. **G2**: (n = 11). Simulated tDCS. **Both groups**: 4 training sessions in a bimanual cooperative task called CIRCUIT, 1 intervention session and 1 retention session per group (1 week apart).	Bimanual speed/accuracy compensation (Bi-SAT). Bimanual coordination factor (Bi-Co). Generalization test: completing a new CIRCUIT design. V_0_: Before treatment. V_1_: 30 min. V_2_: 60 min. V_3_: 1 week.	G1-G2: Patients were able to learn and retain cooperative bimanual skills. G1-G2: No significant differences.
**Picelli** **2019** [24]	40 patients with chronic supratentorial stroke.	Double-blind RCT. **G1**: (n = 20). Cathodic transcranial direct current stimulation over the contralesional cerebellar hemisphere and cathodic transcutaneous spinal direct current stimulation. **G2**: (n = 20). Cathodic transcranial direct current on the ipsilesional cerebellar hemisphere and cathodic transcutaneous spinal direct current. **Both groups**: Robotic gait training. 10 sessions (20 min, 5 days/week, two weeks).	6 MWT. FAC. MI. MAS. Gaitrite® pressure-sensitive walkway. V_0_: Before treatment. V_1_: After the 1st session. V_2:_ 2 weeks. V_3_: 4 weeks.	G1-G2: Both groups showed significant improvements within the group in the 6 MWT test at all time points. G1-G2: No significant differences were found between the groups in 6 MWT, FAC, MI, MAS, cadence, and gait support.
**Alisar** **2020** [18]	32 patients hospitalized for stroke	Double-blind RCT. **G1**: (n = 16). 30 min of bihemispheric tDCS. **G2:** (n = 16). 30 min of simulated bihemispheric tDCS. **Both groups:** 15 sessions (3 weeks) of conventional PT and OT.	FME. FIM. BSSR. V_0_: Before treatment. V_1_: 3 weeks.	G1: Significant improvement in FMUE, FIM and BSSR. G1-G2: Significant improvement in FIM. Most noticeable improvement in stroke with > 6 months of evolution.
**AES**				
**Lee and Lee** **2019** [25]	30 stroke patients.	RCT. **G1**: (n = 15). AES with MT. **G2**: (n = 15). Simulated ESA with MT. **Both groups**: AES (real or simulated combined with MT, 60 min per day, 5 days per week, for 4 weeks.	Motor function with hand dynamometer. MAS. BBS. Gaitrite^®^ pressure-sensitive walkway. V_0_: Before treatment. V_1_: 4 weeks.	G1: Significant differences in all variables. G2: Pre–post treatment improvements, but no significant differences. G1-G2: Significant differences in muscle strength, BBS, walking speed, and stride length and step length.
**FES**				
**Kim and Jang** **2021** [31]	60 patients with chronic stroke.	RCT. **G1:** (n = 20). MT + FES (EMG-FES). **G2:** (n = 20). MT. **G3:** (n = 20). Conservative treatment (CON). **Three groups**: 60 min sessions 5 times/week for 8 weeks.	Biorescue (COP, LOS). BBS. FRT. 10 MWT. V_0_: Before treatment. V_1_: 8 weeks.	G1-G2-G3: The G1 intervention was most effective in COP, LOS, BBS, FRT, and 10 MWT in patients with chronic stroke. G1-G2-G3: In G1, significant differences were obtained in COP, LOS, BBS, FRT and 10 MWT compared to G2 and G3. In patients with chronic stroke, G1 was more effective in all variables studied compared to G3.
**Dantas** **2023** [32]	28 stroke patients > 3 months	Prospective, longitudinal, randomized, crossover study. **G1**: (n = 14). 1°: TT-FES. 2°: TT only. **G2**: (n = 14). 1°: TT only. 2°: TT-FES. **Both groups**: 12 sessions divided into 6 sessions per phase of 30 min, with a frequency of 2/week.	TUG. 10 MWT. FMA. BBS. LEMOCOT. 6 MWT. MMSE. V_0_: Before treatment. V_1_: 6 sessions (3 weeks). V_2_: 12 sessions (6 weeks).	G1-G2: In V_1_ and V_2_, significant improvements in FMA. In V_2_, significant improvements in BBS, LEMOCOT (in non-paretic limb) and 6 MWT. G1-G2: In V_1_, significant improvements in G1 compared to G2 in TUG, BBS, 6 MWT and LEMOCOT. Between V_0_ and V_1_, there were no significant differences between groups. At V_2_, significant differences in G2 compared to G1 in LEMOCOT (paretic limb); and in G1 compared to G2 in TUG. The order of the protocols did not modify the results.

Table created by authors. Description of the intervention procedure in the articles included in the systematic review. Abbreviations: RCT, randomized controlled trial; NMES, neuromuscular electrical stimulation; TENS, transcutaneous electrical nerve stimulation; rTMS, repetitive transcranial magnetic stimulation; tDCS, transcranial direct current stimulation; AES, afferent electrical stimulation; FES, functional electrical stimulation; MT, mirror therapy; RAT, robot-assisted therapy; RFE, repetitive facilitation exercise; MAS, Modified Ashworth Scale; FMA, Fugl-Meyer Assessment; FMA-UE, Fugl-Meyer Assessment for Upper Extremity; ARAT, Action Research Arm Test; BBT, Box and Block Test; MAL, Motor Activity Log; FIM, Functional Independence Measure; BSSR, Brunnstrom stages of stroke recovery; BBS, Berg Balance Scale; FAC, Functional Ambulation Category; MI, Motricity Index; 10 MWT, 10 m walk test; 6 MWT, 6 min walk test; 2 MWT, 2 min walk test; TUG, Timed Up and Go test; FRT, Functional Reach Test; pROM, passive range of motion; COP, center of pressure; LOS, limits of stability; FE, flexion–extension; PS, pronation–supination; AA, abduction–adduction; and EVA, visual analog scale.

**Table 4:** Parameters used in electrotherapy techniques.

Technique	Author	Intervention	Parameters
**NMES**	Baricich 2019 [17]	**G1**: (n = 15) 1 NMES session on injected agonist muscles. 5 NMES sessions on antagonist muscles. **G2:** (n = 15) 1 NMES session on injected agonist muscles. **Both groups**: 10 daily physiotherapy sessions of 60 min 2 weeks (5 days/week).	**G1**: 0.2 ms, 4 Hz, rectangular biphasic balanced current and 0.2 ms, 20 Hz, rectangular biphasic balanced current. **G2:** 0.2 ms, 4 Hz, rectangular biphasic balanced current.
**NMES vs. TENS**	Yen 2019 [26]	**G1:** (n = 13). Transcutaneous nerve stimulation and standard early rehabilitation. **G2:** (n = 13). Neuromuscular electrical stimulation and standard early rehabilitation. **G3**: (n = 14) Standard early rehabilitation. **Three groups**: 10 sessions (30 min per day, 5 days/week, for two weeks).	**G1:** 0.2 ms pulses at 100 Hz, in constant mode and within the participant’s sensory level, without muscle contraction. **G2:** Biphasic square wave, with a pulse duration of 0.3 ms and a frequency of 30 Hz.
**NMES and rTMS**	Etoh 2019 [21]	**G1:** (n = 10). Real NMES + simulated NMES. **G2**: (n = 10). Simulated NMES + real NMES. **Both groups**: 20 sessions over 4 weeks (5 days/week) of NMES (real or simulated) for 10 min, simultaneously with rTMS (1 Hz) in the unaffected motor cortex and RFE for 60 min.	**Real:** Stimulation pulse with triangular waveform and a pulse width of 50 s. Patients received NMES at 1 Hz with a burst of 7 pulses. The intensity of the electrical current was adjusted to produce a slight contraction of the target muscle (16–38.5 mA, maximum voltage of 150 V). **Simulated:** 1 mA. **rTMS:** 600 pulses, 1 Hz, and a stimulation intensity of 90% of the resting motor threshold (rMT).
**tDCS**	Cunningham 2015 [30]	**G1:** (n = 6). Anodal tDCS in ipsilesional upper motor areas. **G2:** (n = 6). Sham tDCS. **Both groups:** tDCS (real or sham) and movement restriction therapy for 30 min, twice daily, 3 days/week for 5 weeks (30 sessions).	**G1:** 1 mA throughout the duration of a rehabilitation session. **G2:** 1 mA, administered transiently (30 to 60 s) at the beginning, then slowly turned off after habituation.
	Allman 2016 [29]	**G1**: (n = 11). Real anodal tDCS. **G2:** (n = 13). Simulated anodal tDCS. **Both groups**: 9 one-hour sessions of tDCS (real or simulated) and motor training on consecutive days.	**G1**: The current was increased for 10 s, maintained at a constant 1 mA for 20 min, and then reduced for 10 s. **G2:** The current was increased for 10 s and then immediately turned off.
	Straudi 2016 [27]	**G1**: (n = 12). Robot-assisted therapy (RAT) + real tDCS. **G2**: (n = 11). RAT + sham-tDCS. **Both groups:** 10 sessions (5 sessions/week), two weeks.	**G1**: Continuous stimulation for 30 min, with an intensity of 1 mA. **G2:** The current was administered for 30 s and then interrupted, but the tDCS device was left in place for 30 min.
	Hamoudi 2018 [28]	**G1**: (n = 18). tDCS (anode over the ipsilesional M1 and cathode on the contralesional forehead). **G2**: (n = 18). Simulated tDCS. **G3**: (n = 14). No treatment. **G1-G2:** tDCS (real or simulated) and sequential isometric pinch strength visual task for 1 h, 5 days a week, for 1 week.	**G1:** 20 min of 1 mA current (current density 0.4 A/m^2^, total charge 0.048 C/cm^2^). **G2:** The current was increased and then reduced for 30 s to induce sensations in the scalp similar to those of real stimulation.
	Beaulieu 2019 [19]	**G1**: (n = 7). Real tDCS. **G2**: (n = 7). Simulated tDCS. **Both groups**: 12 sessions of 60 min upper limb resistance training over 4 weeks (3 days/week).	**G1:** Applied during the first 20 min of each of the 12 sessions at an intensity of 2 mA (with progressive increase/decrease). **G2:** Stimulation only during the first 30 s to ensure adequate blinding.
	Koganemaru 2019 [22]	**G1:** 2 sessions (1 week between sessions). 1 session of oscillatory transcranial direct current stimulation on the affected area of the foot M1 and 1 session of simulated stimulation during treadmill walking. Ankle dorsiflexion was assisted by electrical neuromuscular stimulation in real and simulated conditions.	**G1:** 2 mA sinusoidal wave (from 0 mA to +2 mA) peak-to-peak amplitude.
	Mazzoleni 2019 [23]	**G1:** (n = 20). Real tDCS. **G2:** (n = 20). Simulated tDCS. **Both groups**: 30 sessions of stimulation (real or simulated) and robotic wrist rehabilitation over 6 weeks (5 sessions/week).	**G1:** 2 mA, 20 min, and the anodal electrode in the primary motor cortex area—M1—of the affected hemisphere. **G2:** Simulated stimulation through an activation ramp for 5 s.
	Doost 2019 [20]	**G1:** (n = 10). tDCS. **G2**: (n = 11). Simulated tDCS. **Both groups**: 4 training sessions in a cooperative bimanual task called CIRCUIT, 1 intervention session and 1 retention session per group (separated by 1 week).	**G1:** Actual stimulation began with a gradual increase from 8 s to 1 mA over 30 min and ended with a gradual decrease over 8 s. **G2:** Gradual increase from 8 s to 1 mA over only 30 s of stimulation, followed by a gradual decrease over 8 s.
	Picelli 2019 [24]	**G1**: (n = 20). Cathodic transcranial direct current on the contralesional cerebellar hemisphere and cathodic transcutaneous spinal direct current **G2**: (n = 20). Cathodic transcranial direct current on the ipsilesional cerebellar hemisphere and cathodic transcutaneous spinal direct current **Both groups**: Robotic gait training. 10 sessions (20 min, 5 days/week, two weeks).	**G1:** 2 mA applied for 20 min, with the cathode over the contralesional cerebellar hemisphere. **G2:** 2 mA applied for 20 min, with the cathode positioned over the ipsilesional cerebellar hemisphere. **G1-G2:** The intensity of tsDCS was set at 2.5 mA and applied for 20 min.
	Alisar 2020 [18]	**G1**: (n = 16). 30 min of bihemispheric tDCS. **G2:** (n = 16). 30 min of simulated bihemispheric tDCS. **Both groups**: 15 sessions (3 weeks) of conventional PT and OT.	**G1:** 2 mA for 30 min during the OT session. **G2:** The stimulator was turned on, increasing the current until the patient felt a ‘tingling’ sensation on the scalp for 30 s, then turned off after 1 min.
**AES**	Lee and Lee 2019 [25]	**G1**: (n = 15). AES with MT. **G2**: (n = 15). Simulated AES with MT. **Both groups**: AES (real or simulated combined with MT, 60 min per day, 5 days per week, for 4 weeks.	**G1:** 15 min at 100 Hz and a pulse width of 300 µs and 15 min at 15 Hz and a pulse width of 300 µs. The intensity of the electrical stimulation was adjusted within the range that the patient could perceive. **G2:** The AES device was set to remain inactive.
**FES**	Kim and Jang 2021 [31]	**G1:** (n = 20). MT + FES (EMG-FES). **G2:** (n = 20). MT. **G3:** (n = 20). Conservative treatment (CON). **Three groups:** 60 min sessions 5 times/week for 8 weeks.	**G1:** Current increased for 0.1 s, contracted for 5 s and decreased for 2 s. Stimulation was then performed with a current of 10–20 mA and 35 Hz. To minimize fatigue, a 5 s rest period was allowed between contractions. If the patient did not exceed the reference threshold, electrical stimulation was automatically initiated after 20 s.
	Dantas 2023 [32]	**G1**: (n = 14). 1°: TT-FES. 2°: TT only. **G2**: (n = 14). 1°: TT only. 2°: TT-FES. **Both groups**: 12 sessions divided into 6 sessions per phase of 30 min, with a frequency of 2/week.	**G1-G2:** WalkAide^®^ stimulator (Innovative Neurotronics, Austin, TX, USA) commercially available as a foot drop stimulator. The specific electrotherapy parameters used include a frequency of 35 Hz, an intensity of 10–20 mA, and an adjustable pulse duration according to the patient’s needs.

Table created by authors. Abbreviations: NMES, neuromuscular electrical stimulation; TENS, transcutaneous electrical nerve stimulation; rTMS, repetitive transcranial magnetic stimulation; tDCS, transcranial direct current stimulation; tsDCS, transcutaneous spinal direct current stimulation; AES, afferent electrical stimulation; FES, functional electrical stimulation; EMG-FES, electromyography-triggered functional electrical stimulation; MT, mirror therapy; RAT, robot-assisted therapy; RFE, repetitive facilitation exercise; TT, treadmill training; TT-FES, treadmill training with functional electrical stimulation; M1, primary motor cortex; Hz, hertz; mA, milliampere; ms, millisecond; µs, microsecond; s, second; min, minutes; V, volt; rMT, resting motor threshold.

## Data Availability

All data from the studies are shown in the tables and in the written work.

## Supplementary Materials

The following supporting information can be downloaded at: https://www.mdpi.com/article/10.3390/jcm15083085/s1. PRISMA 2020 Checklist; Table S1: Comparative Summary of Electrotherapy Modalities.

## Abbreviations

The following abbreviations are used in this manuscript:

ECA	Randomized Clinical Trial
NMES	Neuromuscular Electrical Stimulation
V_0_	Assessment Before Treatment
V_1_	First Assessment After Treatment
V_2_	Second Assessment After Treatment
V_3_	Third Assessment After Treatment
10 MWT	10 m Walk Test
MAS	Modified Ashworth Scale
pROM	Passive Range of Motion
FM	Muscle Strength
tDCS	Transcranial Electrical Stimulation
FMA	Fugl-Meyer Assessment
FIM	Functional Independence Measure
BSSR	Brunnstrom Stages of Stroke Recovery
ACV	Cerebrovascular Accident
ARAT	Action Research Arm Test
BBT	Box and Block Test
RFE	Repetitive Facilitating Exercise
6MWT	6-Minute Walk Test
VAS	Visual Analog Scale
FE	Flexion–Extension
PS	Pronation–Supination
AA	Abduction–Adduction
FAC	Functional Ambulation Category
MI	Motricity Index
AES	Afferent Electrical Stimulation
MT	Mirror Therapy
RAT	Robot-Assisted Therapy
MAL	Motor Activity Log
MRI	Magnetic Resonance Imaging
FES	Functional Electrical Stimulation
EMG-FEST	Electromyography-Activated Functional Electrical Stimulation
BBS	Berg Balance Scale
WMFT	Wolf Motor Function Test
TUG	Time Up and Go
LEMOCOT	Lower Extremity Motor Coordination Test
MMSE	Mini-Mental State Examination
FRT	Functional Reach Test
TT	Treadmill Training
